# Social Preferences Toward Humans and Machines: A Systematic Experiment on the Role of Machine Payoffs

**DOI:** 10.1177/17456916231194949

**Published:** 2023-09-26

**Authors:** Alicia von Schenk, Victor Klockmann, Nils Köbis

**Affiliations:** 1 Center for Humans and Machines, Max Planck Institute for Human Development; 2 Department of Economics, University of Würzburg

**Keywords:** machine behavior, cooperative AI, human–computer interaction, social preferences

## Abstract

There is growing interest in the field of cooperative artificial intelligence (AI), that is, settings in which humans and machines cooperate. By now, more than 160 studies from various disciplines have reported on how people cooperate with machines in behavioral experiments. Our systematic review of the experimental instructions reveals that the implementation of the machine payoffs and the information participants receive about them differ drastically across these studies. In an online experiment (*N* = 1,198), we compare how these different payoff implementations shape people’s revealed social preferences toward machines. When matched with machine partners, people reveal substantially stronger social preferences and reciprocity when they know that a human beneficiary receives the machine payoffs than when they know that no such “human behind the machine” exists. When participants are not informed about machine payoffs, we found weak social preferences toward machines. Comparing survey answers with those from a follow-up study (*N* = 150), we conclude that people form their beliefs about machine payoffs in a self-serving way. Thus, our results suggest that the extent to which humans cooperate with machines depends on the implementation and information about the machine’s earnings.

Human interaction with algorithms pervades social life (Doya et al., 2022; Rahwan et al., 2019). Intelligent machines now predict health-care costs (Obermeyer et al., 2019), detect fraud and corruption (Köbis et al., 2022), recommend crime sentences (Kleinberg et al., 2018), and have started to roam the streets in the form of autonomous vehicles (Awad et al., 2018; Bonnefon et al., 2016). Knowingly or unknowingly, humans (will) increasingly interact with intelligent algorithms (Roese & Amir, 2009). Although in the past these interactions with machines have followed a clear script, in that humans command machines to execute tasks predictably, this hierarchical relationship is changing. Thanks to recent developments in artificial intelligence (AI)—particularly machine learning—and growing available (training) data and computing power, artificially intelligent systems are no longer confined to merely executing humans’ commands (Domingos, 2012; Floridi & Sanders, 2004). Such systems produce autonomous, often unpredictable outcomes (Silver et al., 2017) and are thus commonly described as “AI agents” (Floridi & Sanders, 2004; Köbis et al., 2021).

In a growing range of strategic zero-sum interactions, such as common board and computer games, AI agents have surpassed human-level performance (Schrittwieser et al., 2020; Silver et al., 2017). More recently, interest has grown in non-zero-sum, cooperative human–machine interactions, in short, “cooperative AI” (Dafoe et al., 2020, 2021). Evolutionarily, the success of the human species has largely depended on homo sapiens’ unique cooperation abilities (Nowak et al., 2004; Wright, 2001). Introducing AI agents to social life presents the technical challenge of equipping AI systems with compatible capabilities to cooperate with humans (Russell, 2019). Moreover, it presents the challenge of designing AI systems in such a way that psychological roadblocks hindering people’s willingness to cooperate with machines are overcome (Cadario et al., 2021; Shariff et al., 2017).

Striving to meet these challenges requires understanding people’s social preferences toward machines. Because of the immense importance of social preferences for social and economic life (Fehr & Gächter, 2000; Fisman et al., 2015; Thielmann et al., 2020) and the growing social influence of machine behavior on human life (Dafoe et al., 2021; Gambino et al., 2020; Köbis et al., 2021; Rahwan et al., 2019), this question has received much empirical interest across the behavioral and computer sciences (for reviews, see Chugunova & Sele, 2020; March, 2021; Nass & Moon, 2000; Nielsen et al., 2022; Oliveira et al., 2021).

In fact, research on human–machine cooperation can be traced back to the 1980s when Robert Axelrod organized a tournament to determine which algorithm would best perform in a repeated prisoner’s dilemma (Axelrod & Hamilton, 1981). The winning algorithm, submitted by Anatol Rapoport, was the simple tit-for-tat strategy. It prescribes cooperation on the first move and subsequently reciprocates the interaction partner’s previous move. More recently, human–computer interactions have moved from studying such static if-then algorithms that act according to predetermined scripts toward more dynamic (learning) algorithms (Crandall et al., 2018; McKee et al., 2021). Prominent examples of such dynamic AI agents are the reinforcement learning algorithms S++ and S#, which react to the human partners’ moves. They can also engage in cheap talk and surpass human performance in establishing and sustaining cooperation with humans (Crandall et al., 2018; Ishowo-Oloko et al., 2019).

Whether using static algorithms or dynamic AI agents, the aggregated findings from the literature reviews reveal substantial differences in how machine players are introduced as cooperation partners in economic games. One crucial difference pertains to how the payoffs for the machine are implemented.

## Different Implementations of Machine Players

Existing research designs diverge regarding the crucial aspects of (a) what happens to the payoffs earned by algorithms and (b) what information about the payoffs is provided to the participants. We systematically categorized the instructions used in 160 behavioral studies reviewed by March (2021) and found five main ways of such machine-payoff implementations.

The first one is paying the money earned by the machine to a deserving human beneficiary—someone who has exerted effort and was involved in the creation of the algorithm. This implementation has been used in (only) two studies in which the machine players’ payoffs were passed on to the programmer of the algorithm (Azaria et al., 2016; Lin et al., 2014). It thus reflects the financial flow of earnings outside the lab such that companies or individuals behind algorithms benefit from the machine’s actions (Köbis et al., 2021).

Second, another group of seven studies also used a human beneficiary who, however, was not involved in the task. These studies use a so-called token player as the eventual receiver of the machine payoffs (Köbis & Mossink, 2021). For example, Farjam and Kirchkamp (2018) informed participants that a “replaced human receives at the end of the experiment the amount that the computer has earned” (p. 260).

Third, in 14 experiments, participants learned that the machine earns the payoffs. For example, the instructions for a study by Johnson and colleagues (2002) read that the machine is “programmed to make as much money as possible for itself” (p. 45). This implementation reflects the widespread notion of computers as social actors (Nass et al., 1994; Nielsen et al., 2022) and aims to treat them as similar to their human counterparts as possible.

Fourth and relatedly, the experimental instructions of five experiments informed participants that the machine’s earnings would be “burnt.” Although not in a literal sense—destroying money is illegal in many countries—it emphasizes that no human (player) actually earns the payoffs. For example, Cox (2017) instructed participants that “the robot player is computerized and receives no earnings” (p. 114). In both of these implementations, no actual human behind the machine earns the payoffs.

Fifth, 132 studies omitted the information about the payoffs earned by the machines altogether (e.g., Crandall et al., 2018; Ishowo-Oloko et al., 2019). Hence, in this largest category of studies, participants received no information about what happens to the payoffs earned by the machine players.

These different payoff implementations likely influence how people behave when cooperating with machines. Whether a human behind the machine earns the payoffs probably shapes people’s willingness to forgo profit for machine partners. In addition, it is conceivable that people would rather cooperate with machines when they know that a deserving receiver (i.e., the programmer) earns the payoffs rather than an undeserving one (i.e., an uninvolved token player). It is also unclear what beliefs people form and how they subsequently act when people receive no information about the machine’s payoffs.

A comparative analysis of how these different implementations of machine players in economic games influence social preferences is lacking, undermining the understanding of how cooperation with machines differs from cooperation among humans (Dafoe et al., 2020). Moreover, the lack of systematic insight hinders efforts to train algorithms on human preferences (Christiano et al., 2017). In a controlled experiment, we fill this knowledge gap and aim to advance the growing field of cooperative AI by estimating canonical social-preferences parameters toward machines across all existing payoff implementations.

**Box 1.** Paying machines money?

Paying monetary payoffs to a machine reflects a trend in research on human–machine interactions. Recent empirical insights by Makovi and colleagues (2023) substantiated that paying money to machine players provides an adequate proxy for the currencies of real-life human–machine interactions. In their study, participants (*N* = 299) indicated how much they agree that different receivers need or desire currencies like money, with answers given on a scale ranging from 0 (*definitely disagree*) to 100 (*definitely agree*). Participants indicated whether (a) machines need or desire money, (b) machines behave as if they desire money in the sense that they are programmed that way, and (c) humans need or desire money. Their results showed that people generally perceive machines as not desiring or needing money (*M* = 25.8, *SD* = 31.0; significantly lower than the midpoint of the scale, *p* < .001). They also found that people perceive humans to need or desire money (*M* = 82.6, *SD* = 18.1; significantly higher than the midpoint of the scale, *p* < .001). Note that people perceive that machines can behave as if they desire money when programmed to do so (*M* = 64.6, *SD* = 25.8; significantly higher than the midpoint of the scale, *p* < .001). This situation occurs, for instance, when chatbots are programmed with the objective function of extracting payment from their human counterparts (see also Box 2). Although machines experience the reward of receiving money differently than humans, such initial findings confirm that people indeed perceive machine partners as wanting and needing money when they are programmed in that way. To determine how much people take the machines’ needs and desires into account when interacting with them is the main aim of the current study.

## Method and Sample

### Main study: Eliciting revealed social preferences toward machines

Two main categories of social-preference elicitations exist. “Stated preferences” describe what people say they would do. Although stated preferences provide important insights about attitudes and opinions, they can bring methodological challenges—such as experimenter-demand effects (de Quidt et al., 2018)—when behavior toward machines is studied (McKee et al., 2022). Recent evidence in the context of human and machine behavior, for example, suggests that people’s stated intentions do not necessarily match what they actually do when financial consequences are involved (Köbis & Mossink, 2021). “Revealed preferences,” in contrast, refer to what people actually do. Aiming to assess people’s actual behavior toward machines, we thus elicit revealed social preferences. We do so by relying on standardized incentivized economic games with restricted choice sets (Bruhin et al., 2018).

#### Measures to elicit revealed social preferences

As a first measure to elicit revealed social preferences, we use the dictator game, which is a common task in behavioral science. In this task, one person, the “dictator,” is given a sum of money and decides how much, if any, to give to a second person, the receiver. In this task, the receiver is completely passive and does not make any decisions. The dictator game is arguably the simplest economic-decision setting and is therefore often used to study social preferences (toward humans or machines) void of any strategic concerns (Nielsen et al., 2021). In the current study, participants made 30 consecutive binary dictator choices between two different distributions of payoffs. One decision (labeled “Option *X*”) was more favorable to the dictator than the other (labeled “Option *Y*”) in each round (for an overview of the entire decision space of all 30 choices, see Fig. S1 in the Supplemental Material available online). Participants learned that one of the 30 rounds would be randomly chosen and paid out. Thus, there was no need for any feedback between rounds.

**Fig. 1.**
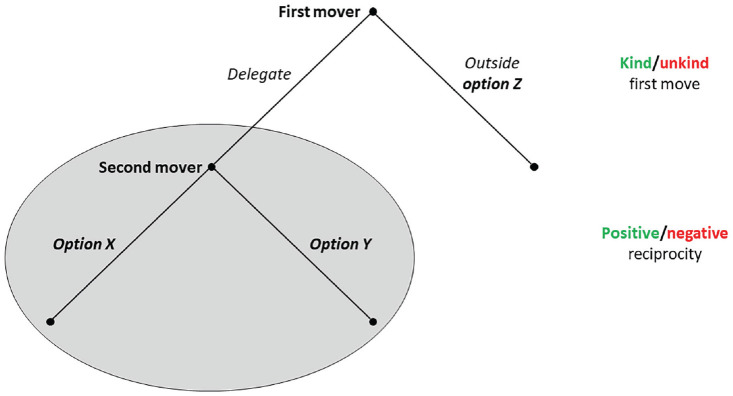
Illustration of the game structure of the reciprocity game used in the main study.

Second, we use the reciprocity game (Bruhin et al., 2018), a two-player game in which the first mover decides between (a) an outside option (Option *Z*) and (b) delegating the decision between two allocation choices (the same set of Options *X* and *Y* as in the dictator game) to the second mover (see Fig. 1). We used the strategy method (Selten, 1967) in which second movers report their intended strategies before the game even if the first mover opts against delegation. This implementation allows studying how participants make decisions for all possible scenarios of how other players behave. Moreover, using the strategy method enabled us to collect data for all possible second movers’ decisions about the two allocation choices, *X* and *Y*, independent of the actually implemented choices by the first mover.

For all pairs of Options *X* and *Y*, we implement two Options *Z*, selected at random. Either the first mover receives a higher payoff and the second mover receives a lower payoff with Option *Z* than with both other alternatives, in which case delegation is a kind move and might trigger positive reciprocity. Alternatively, Option *Z* assigns a higher payoff to the second mover and a lower payoff to the first mover than Options *X* and *Y*, in which case delegation is an unkind move and might trigger negative reciprocity. Again, participants played 30 rounds, out of which one was randomly chosen for payoff. Reciprocity is among the most commonly studied contexts for cooperation with machines (Fogg & Nass, 1997).

Both games are structurally similar in that they entail a binary distribution decision and are played repeatedly for 30 rounds. Moreover, the payoffs of these decisions across rounds are structured in a way that allows us to additionally test how people respond to different forms of inequality of payoffs (see more details in the Results section). The only difference to the dictator game is that in the reciprocity game, there is a choice by the first mover preceding the binary allocation decision.

#### Sample and treatments

We recruited participants from the United States via Prolific Academic (total *N* = 1,198, of which 899 were decision makers; age: *M* = 35.75 years, *SD* = 10.91; 54.73% female). We randomly assigned participants to one of six between-subjects treatments. In one group that served as a baseline treatment, participants played with a fellow human partner (fellow-human treatment). In all other treatments, they played with a machine partner (acting in the role of the receiver in the dictator game and the first mover in the reciprocity game).

**Fig. 2.**
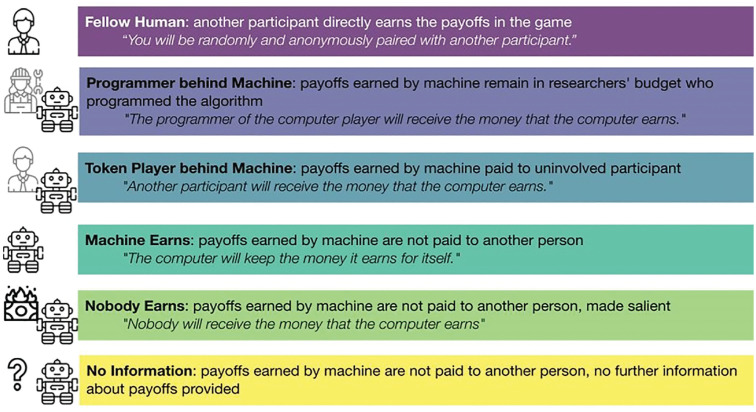
Overview of the different between-subjects treatments used in the main study.

We implemented this machine player in different ways. Participants learned that the payoffs either (a) were transferred to the programmer of the algorithm (programmer-behind-machine treatment), which in our case was one of the experimenters, who kept the payoffs for the research budget; (b) were transferred to an independent and uninvolved third person, that is, another participant (token-player-behind-machine treatment); (c) remained with the machine and were thus effectively not paid out (machine-earns treatment); or (d) were paid to no one (nobody-earns treatment). In (e) the no-information treatment, participants received no further information about the machine’s payoffs. Figure 2 presents an overview of these treatments that manipulate the counterpart type and the payoff implementation with the most important statements from the instructions.

Although in the fellow-human, programmer-behind-machine, and token-player-behind-machine treatments the final payoffs are identical (= a human earns), adding a machine as an intermediary between the giver and the eventual receiver could affect people’s willingness to share their endowment. In particular, machine intermediaries might increase the psychological distance from the receiver and make people share less.

We conducted the study in a fully incentivized way. Each participant played one type of game in one treatment to avoid carryover effects between the games. In the fellow-human and the token-player-behind-machine treatments, we recruited participants to passively receive the (machine) payoffs. In the programmer-behind-machine treatment, we transferred the payoffs to the researchers’ budget because they programmed the algorithm of the machine player (for a more detailed description, see the Machine Play section). For each treatment, we recruited 50 decision makers for the dictator game and 100 for the trust game (twice as many as for the dictator game because of the two different Options *Z* per pair of Options *X* and *Y*). On average, participants earned £2.23 for a study that lasted about 9 min.

#### Demographics and exit questions

After participants engaged in the economic games, we assessed standard demographic information regarding age, gender, the highest level of education, and, if applicable, the field of study. With single items on 5-point scales, we further assessed the participants’ familiarity with new technologies (0 = *not familiar at all*, 4 = *very familiar*), confidence in new technologies (0 = *no confidence at all*, 4 = *strong confidence*), and stated preferences for redistributive policies by the government (0 = *redistribution should be decreased a lot*, 4 = *redistribution should be increased a lot*). We report comprehensive analyses of how these measures correlate with social preferences in the appendix and Table S7 in the Supplemental Material. We also elicited participants’ beliefs about what happens to the payoffs using an open text box.

### Machine play

The machine players were programmed to imitate human behavior in the role of the first mover in the reciprocity game. Using the behavioral and demographic data from the fellow-human treatments, we trained a classification algorithm to predict choices from personal characteristics. We then drew an “artificial” human using the distribution of demographic information in the fellow-human subject population (for more details, see the appendix in the Supplemental Material). We predicted how this individual would have decided by training a classification algorithm to make 30 out-of-sample predictions on the basis of demographic information and stated views about AI.

### Ethics and open-science statement

All data were collected under ethics approval and after obtaining informed consent. All materials, data code to reproduce all analyses, and figures are openly available at https://osf.io/efmvh/?view_only=fbba5877d7014052adbbf404703eb1ff. The experiment programmed in oTree is available at https://github.com/aliciavs/social_preferences_towards_humans_machines.

## Results

### Allocation decisions

**Fig. 3.**
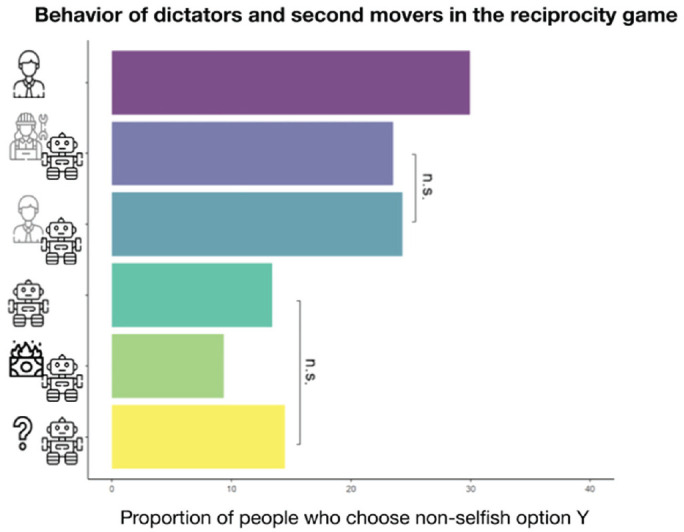
Overview of the proportion of participants who shared with their counterparts in the dictator game and as a second mover in the reciprocity game across treatments. All pairwise comparisons are significant (Tukey’s range test) except for those marked with a bracket.

The first main insight into revealed social preferences toward humans and machines stems from the allocation decisions of the dictator in the dictator game and from the second mover in the reciprocity game (see Fig. 3). It reveals that the proportion of participants who shared with a human (29.93%) was significantly higher than participants who shared with machines. All pairwise comparisons of the share of Option *X* using a Tukey’s range test between the fellow-human treatment and all other treatments are significant. We estimated Cohen’s *d*s of 0.070 and 0.097 in absolute value for the comparison with token-player-behind-machine treatment (24.31%) and programmer-behind-machine treatment (23.51%), respectively, and > 1 for the comparison with the machine-earns (13.42%), nobody-earns (9.36%) and no-information (14.47%) treatments.

Moreover, the description of the machine payoffs made a meaningful difference. The proportion of participants sharing is significantly higher when a human behind the machine (token player or programmer) earned the machine’s payoffs than when no human behind the machine earned the payoffs (machine-earns and nobody-earns treatments), all pairwise comparisons *p* < .01 (Cohen’s *d*s > 1.1 in absolute value). Compared with human–human settings, playing with a machine with no human beneficiary reduced the proportion of nonselfish choices by two thirds, conceptually replicating previous studies that found a strong reduction of dictator-game giving toward computer players (Nielsen et al., 2021).

Overall, we found a three-stepped pattern of results: The likelihood of choosing the nonselfish option was highest toward fellow humans, followed by humans behind machines, and lowest toward machines without any human beneficiary. Moreover, sharing rates by participants who received no information are statistically indistinguishable from the machine-earns treatment (*p* = .984).

Given the variation in the decision space of alternative Options *X* and *Y*, we were further able to take a closer look at altruism and efficiency concerns. The data revealed that only a very small fraction of participants (3%–5%) chose the nonselfish option when there was no human beneficiary and that Option *Y* was not efficient and assigned strictly higher payoffs to the receiver than Option *X*. This behavior most likely stemmed from confusion (Burton-Chellew et al., 2016; Nielsen et al., 2021) or reputation concerns toward the experimenter. Furthermore, Table S1 in the Supplemental Material reports the frequency of choosing Option *X* in the three efficiency scenarios: either Option *X* or Option *Y* being efficient or both options being equally efficient. The observed behavioral patterns indicate that participants still had efficiency concerns toward a machine even if no human beneficiary existed.

### Social-preference-parameter estimation

Besides giving rates in the dictator game and the reciprocity game, we estimated revealed social preferences using the extension of Bellemare and colleagues (2011) of the model by Charness and Rabin (2002). We relied on McFadden’s (1980) random-utility model, which has been previously used in our setting to estimate the social-preference parameters θ = (α, β, γ, δ) (Bruhin et al., 2018). These represent the relative weight the decision maker puts on the other player’s payoffs in different situations. This method assumes that the utility consists of a deterministic utility (behavioral model) and a random component representing noise in the utility evaluation. By exploiting the binary decision structure in both games, the assumption is that a participant chooses Option *X* if the random utility exceeds the utility of Option *Y* to estimate revealed social-preference parameters. We used maximum likelihood estimations to estimate these parameters.

Such a structural model of social preferences has the advantage that it can capture preferences for the distribution of payoffs between the players and the preferences for reciprocity (Bruhin et al., 2018; Charness & Rabin, 2002). The relative size of the estimated parameters from multiple binary decisions also indicates the relative importance of the different aspects of social preferences, thereby allowing richer insights into the multifaceted concept of social preference than merely using choices in one-shot economic games.

The first set of parameters (a) examines how people weigh others’ payoffs under unequal conditions. The parameter α refers to the weight a participant places on the partner’s payoffs for disadvantageous inequality, that is, when the participant’s payoffs are lower than the partner’s payoffs. The parameter β denotes the weight placed on the partner’s payoffs during advantageous inequality, that is, when the participant’s payoffs are higher than the partner’s payoffs.

We estimated the parameters according to:

$$\begin{array}{l} \;\;\;\;\;\;\;\;\;\;u_{1}=\left(1-\alpha s-\beta r-\gamma q-\delta v\right)\pi_{1}+\left(\alpha s+\beta r+\gamma q+\delta v\right)\pi_{2}\\ \;s={\{}_{0\;\;otherwise\;}^{1\;\;if\;\pi_{1}\;<\pi_{2}}\;q={\{}_{0\;\;otherwise}^{1\;\;if\;player\;2\;behaved\;kindly}\\ \;\;r={\{}_{0\;\;otherwise}^{1\;\;if\;\pi_{1\;}>\pi_{2}}\;\;\;\;\;\;\;\;\;\;v={\{}_{0\;\;otherwise}^{1\;\;if\;player\;2\;behaved\;unkindly} \end{array}$$

with π_1_ and π_2_ being the payoffs of Individuals 1 (the decision maker) and 2, respectively; α indicating the weight on the other’s payoffs when behind; β indicating the weight on the other’s payoffs when ahead; γ being a measure of positive reciprocity; and δ being a measure of negative reciprocity (for more details, see the positive and negative reciprocity section).

#### Behindness and aheadness aversion

Our results in the fellow-human treatment largely replicate previous research in that the estimate of α is relatively small (α = −0.07), albeit slightly negative (Charness & Rabin, 2002). In line with previous work (Bruhin et al., 2018; Charness & Rabin, 2002; Klockmann et al., 2022a, 2022b), our estimate for β is much larger than the α estimate (β = 0.35). Put into perspective, one can calculate people’s willingness to increase the other’s payoffs by £1 as follows: When the participant is ahead of the partner and *π*_2_ increases by 1, the utility *u*_1_ increases by β. To make the individual indifferent between giving *π*_2_ and *π*_2_ + 1 to the receiver, one needs to decrease *π*_1_ by β / (1 – β) = £0.53. When behind, however, this amount is slightly negative, α / (1 – α) = −£0.06, indicating that people are not willing to give up money to improve their partner’s payoffs (or are even willing to give up £0.06 of their own payoffs to destroy £1 of the partner’s payoffs).

**Fig. 4.**
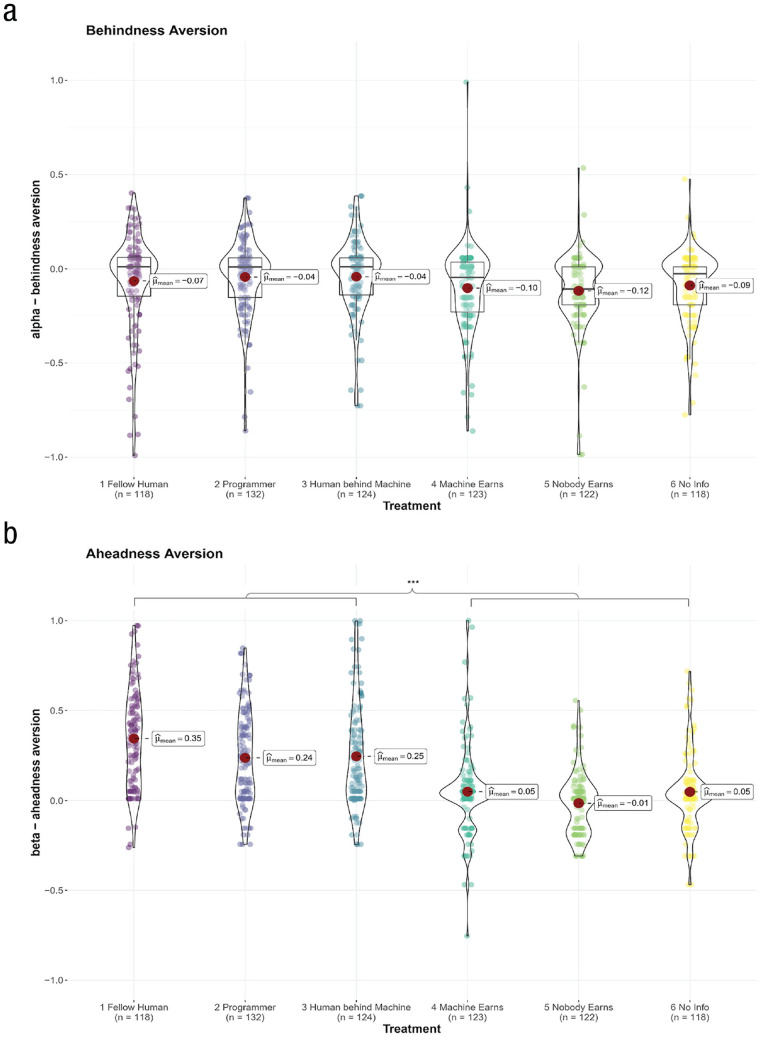
Estimated social preference parameters of (a) a behindness aversion and (b) β aheadness aversion. Asterisks indicate ***p < .001 according to one-way analysis of variance with Holm-Bonferroni correction for multiple comparisons. All pairwise comparisons of the treatments below the left square bracket (human, programmer behind machine, and token player behind machine) and the right square bracket (machine earns, nobody earns, and no information) are significant.

When comparing a and β across treatments, we found that the estimate for a remains stable around 0 (–0.12 ≤ a ≤ 0.04) when playing with machines. Hence, people are equally behindness averse when playing with humans and machines (see Fig. 4a).

A different picture emerges for aheadness aversion—hence advantageous inequality for the human player. Akin to human–human cooperation, people also show aversion to being ahead when playing with humans behind machines (token player: β = 0.24; programmer treatment: β = 0.25). Put into perspective, in such settings with a human beneficiary of machine play, participants are willing to give around £0.31 to improve the partners’ payoffs by £1. However, when cooperating with machines and no human earns the payoffs or when no information is provided, β is significantly smaller and effectively drops to 0 (–0.01 ≤ β ≤ 0.05, see also Fig. 4b; pairwise *p*s < .001, Cohen *d*s > 0.77 in absolute value). Hence, in disadvantageous settings, positive other-regarding preferences disappear when cooperating with machines void of a human beneficiary.

The results for the a and β estimates indicate that people are generally behindness averse independent of whether they interact with humans or machines. These findings extend previous work by showing that the general envy toward machines does not depend on the realization of the payoffs (De Melo et al., 2016). In line with the literature showing that people do not feel guilty about exploiting machines, we also discover that people show aheadness aversion only when cooperating with humans or humans behind machines, not when matched with machines without a human beneficiary.

#### Positive and negative reciprocity

The second set of social-preference parameters concerns reciprocity. Whereas *γ* denotes how many participants help people who helped them (i.e., positive reciprocity), δ denotes how many participants harm people who harmed them (i.e., negative reciprocity).

In the fellow-human treatment, our results replicate previous findings that people generally show positive reciprocity (g = 0.13). Hence, kind acts by fellow human partners increased the weight that participants placed on their payoffs.

In contrast to previous studies, however, we found a positive d of 0.10. To explain this somewhat surprising finding, we examine the structure of the reciprocity game. Because of the binary setting, the prior move is always either unkind or kind but never none of these. Therefore, the small positive d (especially in light of a larger, positive g) indicates a general positive weight on the other player’s payoffs and not necessarily a rewarding or direct positive reaction to an unkind move. For human partners, this means that people appear to be particularly forgiving when faced with an unkind action by the first mover.

**Fig. 5.**
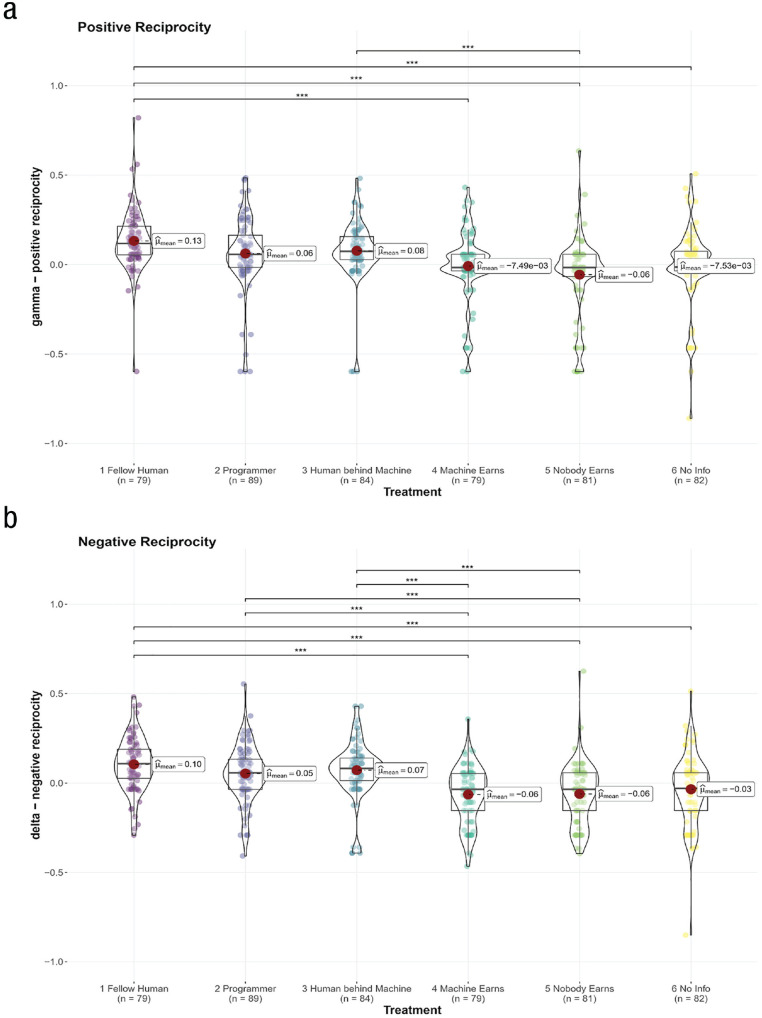
Estimated social-preference parameters of (a) g positive reciprocity and (b) d negative reciprocity across all treatments. Asterisks indicate ***p < .001 based on one-way analysis of variance with Holm-Bonferroni correction for multiple comparisons. All comparisons of the human treatment and the token-player-behind-machine treatment with all treatments under the lower two square brackets (machine earns, nobody earns, and no information) are significant. The group-wise comparisons of the programmer-behind-machine treatment and the treatments under the upper squared bracket (machine earns and nobody earns) are significant.

When comparing reciprocity preferences toward humans and machines, again, we found no differences in settings in which humans eventually earn the payoffs of the machine, for neither positive reciprocity nor negative reciprocity. However, when people cooperate with machines and no human earns the payoffs or no information is provided about the payoffs, a different picture emerges. People showed significantly less positive reciprocity (*p*s < .001 and Cohen’s *d*s > 0.71 in absolute value when comparing the fellow-human treatment with all three treatments). People also showed less negative reciprocity (Cohen’s *d*s > 0.50 in absolute value when comparing one of the three treatments involving a human beneficiary with one of the other three treatments; *p*s < .001 for all comparisons except for programmer vs. no information). In fact, in these treatments, reciprocity disappeared altogether (–0.06 ≤ g ≤ 0.007, –0.06 ≤ d ≤ –0.03; see Fig. 5).

In conclusion, the results of the main study indicate that people show signs of reciprocity toward fellow humans and machines in settings where humans earn the payoffs. Yet reciprocity disappears when there is no human beneficiary or a void of information about the payoffs. Hence, we add nuance to the previously observed general finding that people reciprocate machines (Pratt et al., 2007; Sandoval et al., 2016) by highlighting that such reciprocity depends on the realization of the machine payoffs.

### Clusters of social-preference types

Next, we used a finite mixture-estimation model that endogenously identifies different preference types in our sample. This approach’s key advantage is that it does not require prespecifying the preference types or even the number of types. Here, we assumed that the population comprises *K* distinct preference types, each characterized by its own set of parameters θ(*k*). We then split participants into these *K* > 1 groups and estimated the parameters and size for each group separately.

The analysis revealed that a model with three main behavioral types best fits our data (for a complete overview, see Tables S2 and S3 and the appendix in the Supplemental Material). First is a “mildly altruistic/selfish” type that comprises 28.10% of all participants. It is characterized by sharing relatively little in the dictator game. This group also showed positive aheadness and behindness aversion. Hence, people are concerned about falling behind or being ahead compared with others. This group showed no signs of either positive or negative reciprocity.

The second behavioral type can be described as “spiteful” and accounts for 30.99% of the sample. It revealed strong negative aheadness and behindness aversion, hence a strong preference for being ahead of others and avoiding falling behind. In addition, this group showed no significant signs of positive or negative reciprocity.

The third type is “aheadness averse and positively reciprocal,” hence people who dislike unfair outcomes that favor themselves and answer kind acts with kindness. This type describes 40.91% of the participants.

**Fig. 6.**
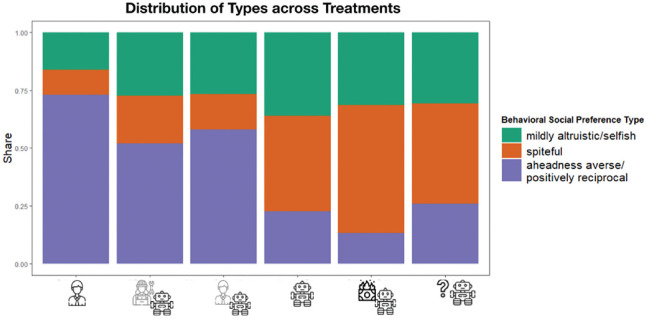
Distribution of behavioral types identified by finite mixture estimations across treatments.

Besides these general classifications, our design allowed us to assess how these behavioral types differ depending on their interaction partner. When interacting with humans, almost three quarters of participants (73.15%) are classified as the aheadness-averse/positive reciprocal*-*behavioral type. When interacting with humans behind machines, this behavioral type still constitutes the majority, albeit to a lesser amount (programmer behind machine = 52.00%; token player behind machine = 58.00%). Note that the proportion of spiteful individuals increases from 10.74% (fellow humans) to 15.33% when a token player earns the payoffs and to 20.67% when the algorithm programmer earns the payoffs (see Fig. 6).

Note that the spiteful type becomes the largest proportion of settings in which people cooperate with machines without a human beneficiary. When the machine earns the profits, the share of spiteful individuals increases to 41.33%, and when it is explicitly stated that nobody receives the machine’s payoffs, it even amounts to 55.33%. The percentage of mildly altruistic/selfish types, although fluctuating between 16.11% and 36.00%, remains the second-largest group across all treatments. Finally, the pattern of behavioral types not receiving any information mimics that of participants being told that the machine earns the payoffs most closely.

**Box 2.** Where do people think the machine’s money goes?

Outside of experimental studies, the money a machine earns does not just disappear but is typically earned by companies that develop and employ the machines. However, companies implementing machine partners like robots, interactive AI systems, and chatbots differ in their approach to disclosing how they benefit from payoffs. Two examples highlight this difference. First, the personalized chatbot Replika has accrued more than 8 million users with the promise to be “the AI companion that cares” and entails a premium version (see website at Replika.ai). When asking a newly created Replika whether one should purchase the premium version, it actively encourages the user to do so. It indicates that it, the chatbot, personally benefits from such payment. Second, a different implementation exists for the popular chatbot ChatGPT, released in 2022 by OpenAI. It, too, features a premium version called “ChatGPT Plus” (see https://openai.com/blog/chatgpt). When asked the same question whether to purchase the premium version, it responds very differently from Replika. It effectively avoids giving a direct recommendation and clarifies instead that the payments do not “personally” benefit the chatbot. There is, however, no explicit information about the company OpenAI behind ChatGPT earning money. Apart from these examples, many companies do not disclose any further details about being the eventual beneficiary of machine payoffs.

### Belief formation in the no-information treatment

After completing the game, participants in our study indicated their beliefs about what happens to the machine payoffs in all treatments in an open-text box. These beliefs are particularly revealing in the no-information treatment in which participants received no information about the actual implementation of the machine payoffs. A research assistant naive to the study’s purpose coded the answers in the no-information treatment according to five categories.

**Fig. 7.**
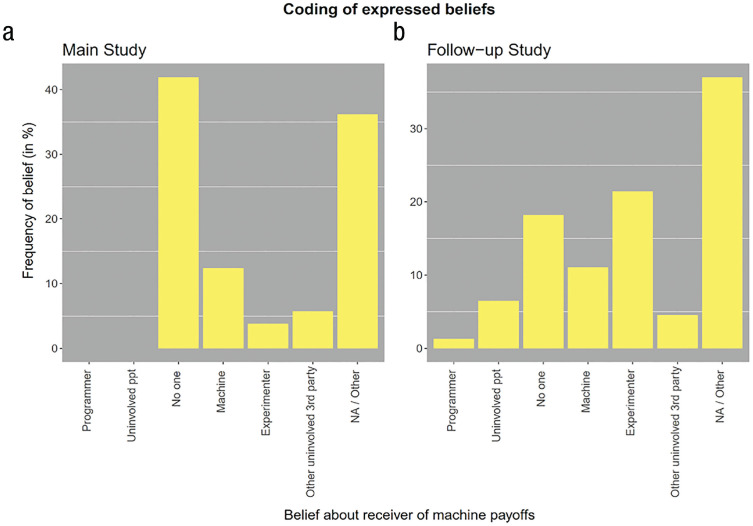
Frequencies of coding for the no-information treatment in the main study and in the follow-up study. The *y*-axis plots the relative frequencies for the coding of the expressed beliefs by a naive research assistant. In the main study, (a) participants engaged in the incentivized tasks and then indicated their beliefs about what happens to the machine payoffs. In the follow-up study, (b) participants read the same instructions but did not engage in the incentivized task and merely indicated their belief about the machine payoffs. The *x*-axis shows the coding scheme of possible receivers of the machine payoffs: “the programmer/company behind the algorithm”; “another uninvolved person”; “no one; the money is not paid out”; “earned by the machine (to use it further)”; “the experimenter”; “another uninvolved third party”; “NA / Other.”

Figure 7a displays the frequencies of the respective answer codings. As shown, the modal answer coding in the main study is “No one; the money is not paid out,” with 41.9% of responses indicating the belief. This belief coincides with the payoff implementation that most strongly facilitates the justification of self-serving behavior. Only very few participants (3.81%) believed the payoffs would remain with the experimenter.

### Follow-up study: Belief formation among uninvolved participants

To examine to what extent these beliefs might be motivated by participants “wanting to believe” that the payoffs disappear because it facilitates the justification to act more selfishly, we conducted a follow-up study. We recruited a sample of 150 U.S. participants from Prolific Academic (age: *M* = 37.21 years, *SD* = 10.26; 35.62% female) who did not engage in the main study. These participants read the same instructions used in the main study and were informed that these instructions had been shown to previous participants. After the control questions, they were asked directly to indicate their beliefs about what happens to the machine payoffs (i.e., they did not engage in the incentivized tasks themselves). We showed the instructions of the dictator game to 50 participants and those of the reciprocity game to 100 participants without providing any information about what happens to the machine payoffs, hence identical to the no-information treatment of the main study.

We asked a new research assistant naive to the study’s purpose to code the answers using the same coding scheme as in the main study. The results of these additional analyses are shown in Figure 7b. Among uninvolved participants in the follow-up study, a much smaller fraction believed the money was not paid out at all (18.18%). A χ^2^ test with Yates’s continuity correction comparing the proportion of people who believe the money was not paid out in the main study and the follow-up study, respectively, revealed a significant difference, χ^2^(1) = 16.34, *p* < .001. In addition, the belief that the money stayed with the experimenter was much more frequent in the follow-up study (21.42%) compared with the main study (3.81%). This difference across studies is significant as well, χ^2^(1) = 14.42, *p* < .001.

## Discussion

The study provides the first systematic and nuanced insights into social preferences toward machines. How revealed social preferences toward machines differ from interhuman social preferences depends strongly on the machine-payoff implementation and the type of social preferences.

For allocation decisions, participants showed the highest levels of sharing propensity when sharing with fellow humans. Choosing the nonselfish option significantly dropped when matched with a machine receiver that had a human beneficiary behind it. We observed this change in decisions in all three treatments, even if a human eventually earns the machines’ payoffs. The mere inclusion of a machine as an intermediary reduced people’s willingness to share their endowment. This matches previous research that showed such intermediaries can lead to different, often less prosocial behaviors (Hsee et al., 2003; Mazar et al., 2008).

Altruistic giving was further reduced for machine counterparts without any human beneficiary. Although previous work has shown the basic pattern of lower altruistic preferences for machines versus humans (Burton-Chellew et al., 2016; Nielsen et al., 2021), we showed that a human beneficiary “behind the machine” can increase giving rates compared with settings without a human beneficiary.

Analyzing the full set of social-preference parameters revealed that the degree to which people differentiated between humans and machines varied according to the specific parameter. For behindness aversion (α), we found no differences whatsoever. This shows that people generally dislike being behind, independent of their partner. In contrast, for aheadness aversion (β), we found that people were averse to being ahead of fellow humans and machines when humans earn the payoffs. Yet people did not seem to mind an advantageous inequality in their favor that comes at a cost for a machine without a human beneficiary. Estimates for positive (γ) and negative (δ) reciprocity revealed a similar pattern: People were sensitive to paying back humans and machines with a human beneficiary. However, this reciprocity norm did not extend to machine partners without a human beneficiary.

Our finite mixture-estimation model identified three types of preferences. The distribution of these types differed starkly across treatments. When interacting with machines, most people disliked favorable inequality and showed positive reciprocity, reflecting typical behavior when interacting with (unknown) others. Yet when interacting with machines and no human earned the payoffs, most people acted spitefully. It adds credence to the notion that people change their behavioral profile contingent on their counterparts, as research on conditional cooperation has shown (Burton-Chellew et al., 2016; Fischbacher et al., 2001). Here, we found evidence that people also adjust their behavior according to different implementations of machine partners. Future research adopting a within-subjects design could help to gain deeper insight into the intraindividual variance of social preferences toward machines.

### Methodological and societal implications

A first methodological implication for the growing field of behavioral research on human and machine cooperation is that machine partners in economic games require close attention. A machine player by no means always eliminates social preferences as they were originally intended to (Andreoni, 1995; Houser & Kurzban, 2002). In our study, social preferences mostly vanished in the treatments in which the payoffs were not paid out at all (no one earns) or paid to the machine (machine earns), which both resulted in the partner’s payoffs being effectively zero. In line with this view, the estimated parameters of aheadness and behindness aversion, reciprocity, and fair-mindedness are all close to zero on average. Besides social preferences, also efficiency concerns would predict not giving in these cases—and indeed, the majority of decisions aligned with that. In these treatments, we found that only a small fraction of people forwent their own payoffs to benefit their partner or to destroy their payoff. Previous research, however, has shown that people even reveal social preferences toward machines when they are fully aware of the game’s logic (Nielsen et al., 2021). A plausible explanation receiving initial empirical support reads that people often act toward machines as if they were humans (Nass et al., 1994; Nielsen et al., 2022). In our study, this tendency was observed for a large fraction of participants only when a human behind the machine earned the payoffs.

Second, the way in which these machine players are incorporated strongly influences how people reveal social preferences toward them. So far, researchers have diverged substantially in how they implement (the payoffs of) machine players in economic games. Here, we provide a comparative overview of the consequences of these design decisions.

So far, the approach most commonly used has been not to provide any information about the machine’s payoffs. Our findings show that this implementation consistently leads to weak revealed social preferences toward machines. In the no-information settings, people acted as selfishly as they did in the treatments in which the money was paid to a machine or explicitly not paid out at all.

When providing no information, people are free to form beliefs about what happens to the payoffs. Answers in the open-text field in our main study regarding beliefs about the machine payoffs suggest that most people, in fact, believe the money is not paid out. In the follow-up study, in which people had no motivation to adhere to this particular belief, the proportion dropped significantly. Hence, our results indicate that the information gap appears to allow people to form motivated beliefs about the payoffs. In line with belief-based utility (Loewenstein & Molnar, 2018), people in the main study (vs. the follow-up study) had an incentive to believe that no human behind the machine earned the payoffs because this setting enabled them to justify acting more selfishly, possibly because it dampened the guilt of selfishness. Because providing no information is the most common implementation in behavioral science, studies without payoff information might lead to weak social-preference estimations. In addition, outside of controlled experimental settings, people often interact with machines without knowing who earns the payoffs in real life (see also Box 2). Our findings align with previous research on human–human cooperation that showed prosocial behavior drops when uncertainty about the outcome of one’s actions exists (Kappes et al., 2018). Lack of information and thus uncertainty about an eventual human beneficiary might lead people to cooperate less with machines compared with when the actual (human) beneficiary is disclosed.

Previous research on human and machine partners has uncovered a transparency–efficiency paradox (Ishowo-Oloko et al., 2019). When people know they interact with a machine (vs. a human), they cooperate less. Note that participants received no information about the machines’ payoffs in these experiments. Paradoxically, transparency (about a human beneficiary of the machines’ earnings) might help to resolve the transparency–efficiency paradox.

Whether a deserving or undeserving human earns these payoffs seems less relevant. For none of the social-preference indicators, we found no significant difference between the setting in which an uninvolved token player or the algorithm programmer earned the payoffs. The machines acting as intermediaries might have mooted the well-established deservingness heuristic (Petersen et al., 2011). This explanation aligns with theoretical work that has argued machines increasingly acting as intermediaries and delegates might reduce human social and moral concerns (Gratch & Fast, 2022; Köbis et al., 2021). Another plausible explanation is that participants perceive uninvolved participants who, like them, are recruited from an online click-work platform like Prolific as equally deserving as the programmer. This notion receives support from empirical research that showed online participants treat other participants from the same online platform preferentially (compared with random strangers; Almaatouq et al., 2020). Whether the lack of differences stems from the imputed deservingness heuristic through machine intermediaries or from platform-based in-group favoritism is an open question for future research to resolve.

Our study focused on one-shot dyadic settings, reflecting many real-life interactions between humans and machines. Consider, for example, industrial settings, in which workers are cooperating one on one with increasingly intelligent machines (Villani et al., 2018); traffic, in which, ever more often, human drivers need to cooperate with autonomous vehicles (Schwarting et al., 2019); or another context that recently gained much attention, human interactions with chatbots (Stokel-Walker & Van Noorden, 2023). So far, most of these machines form no (lasting) memories of previous interactions, effectively rendering each interaction to a one-shot encounter. However, AI systems imbued with more lasting memory capacities are beginning to appear (Saravanan et al., 2022), enabling repeated interactions with AI systems, which is a key step toward turning them into companions (Krämer et al., 2011). Hence, studying the role of machine payoffs for revealed social preferences in such repeated settings presents an important next step for research on human and machine behavior. In addition, moving from one-to-one to one-to-many interactions, such as human interaction with multiple bots on social media, in which behavior is observable provides additional extensions to this emerging line of research.

### Conclusion

The field of research using machine players is evolving. Although machine players were initially introduced to eliminate social preferences, researchers now seek to understand social behavior in interaction with machines. Hence, machine players are no longer a mere research method but are increasingly becoming actors within human social life. Thus, understanding social preferences toward machines marks an increasingly important step toward achieving cooperative AI (Dafoe et al., 2020). Here, we present a systematic behavioral investigation revealing that people’s preferences toward machines differ depending on the machines’ payoffs and the information provided about them. People reveal systematically higher social preferences toward machines when they know that a human beneficiary receives the payoffs accrued by the machine compared with when they know that no such human behind the machine exists. Not informing people about the machine payoffs leads to low social preferences as people form beliefs about the payoffs in a self-favoring way.

## Supplemental Material

sj-docx-1-pps-10.1177_17456916231194949 – Supplemental material for Social Preferences Toward Humans and Machines: A Systematic Experiment on the Role of Machine Payoffs Supplemental material, sj-docx-1-pps-10.1177_17456916231194949 for Social Preferences Toward Humans and Machines: A Systematic Experiment on the Role of Machine Payoffs by Alicia von Schenk, Victor Klockmann and Nils Köbis in Perspectives on Psychological Science

## Transparency

*Action Editor:* Mirta Galesic

*Editor:* Interim Editorial Panel

*Author Contributions*

A. von Schenk and V. Klockmann share first-author status.
