# Supplementary material for: Social Preferences Toward Humans and Machines: A Systematic Experiment on the Role of Machine Payoffs
Source: Perspect Psychol Sci. 2023 Sep 26;20(1):165–81. doi: 10.1177/17456916231194949 (PMC11720266; doi:10.1177/17456916231194949)
Supplement: sj-docx-1-pps-10.1177_17456916231194949 – Supplemental material for Social Preferences Toward Humans and Machines: A Systematic Experiment on the Role of Machine Payoffs [file sj-docx-1-pps-10.1177_17456916231194949.docx]

Figure S1.

Illustration of the decision space for the repeated dictator game choices. Each circle represents ten binary dictator games by a line that connects option X (blue square) and option Y (red dot). The slope of each line represents the cost for the dictator to increase the receiver’s payoffs (option X Pareto-dominant, receiver indifferent, dictator indifferent, conflict of interest). In the top-left (bottom-right) circle, both options in each game represent disadvantageous (advantageous) inequality for the dictator. In the middle circle, the relative position depends on the chosen option. Figure adapted from [Bruhin et al. (2018](#bib8)).

Figure S2.

Illustration of the decision space for the repeated reciprocity game choices. Option X and option Y are depicted as in Figure S1, slope and circles again represent the cost for the second mover to increase the first mover’s payoffs and advantageousness, respectively. The green crosses depict the outside option Z the first mover can choose. In Panel A (B), option Z always assigns less (more) payoff to the second mover than option X and option Y and more (less) payoff to the first mover, thus making delegation a kind (unkind) move triggering positive (negative) reciprocity.

Figure S3.

Frequencies of coding for all treatments in the main study. The y-axis plots the relative frequencies for the coding of the expressed beliefs by a naive research assistant. The x-axis shows the coding scheme of possible receivers of the machine payoffs.

Figure S4.

Bar chart depicting the count distribution of different payoff implementations and information provisions in the papers reviewed by [March (2021).](#bib45)

Figure S5.

Overview of individual estimates of aheadness and behindness aversion, comparing human cooperation partners with all other cooperation partners.

Figure S6.

Overview of individual estimates of positive and negative reciprocity, comparing human cooperation partners with all other cooperation partners.

Figure S7.

Estimated social preference parameters of (A) 𝜑 fair-mindedness and (B) 𝜌 equality–efficiency trade-off across all treatments. Significance coding: * *p* < .05; ** *p* < .01; *** *p* < .001 based on one-way ANOVA with Games-Howell pairwise comparisons and Holm-Bonferroni corrections for multiple comparisons. All comparisons of the Fellow Human treatment and the Token Player behind Machine treatment with all treatments under the lower two in squared brackets (Machine Earns, Nobody Earns, and No Information) are significant. The group-wise comparisons of the Programmer behind Machine treatment and the Nobody Earns treatment are significant. The figure plots the statistical results for the full sample (i.e. not excluding any cases) but “zooms in” on the values in the range of 𝜌 >-10<10.

Table S1.

Overview of likelihood of choosing option X across treatments and efficiency.

|  | Efficient Options | | | Total |
| --- | --- | --- | --- | --- |
| Treatments | None | X | Y |  |
| Fellow Human | 76.73% | 90.32% | 52.53% | 70.07% |
| Programmer behind Machine | 81.78% | 93.83% | 61.56% | 76.49% |
| Token Player behind Machine | 82.67% | 92.72% | 60.67% | 75.69% |
| Machine Earns | 91.11% | 94.17% | 79.60% | 86.58% |
| Nobody Earns | 96.22% | 95.50% | 85.64% | 90.64% |
| No Information | 91.11% | 92.78% | 78.62% | 85.53% |

**Table S2.**

Overview of classification of different behavioral types.

|  | Type 1  **mildly altruistic/ selfish** | Type 2 **spiteful** | Type 3  **aheadness averse/ positively reciprocal** |
| --- | --- | --- | --- |
| Share of X choices | 0.281***  (0.016) | 0.310***  (0.017) | 0.409***  (0.017) |
| α | 0.049***  (0.004) | -0.385***  (0.044) | 0.012  (0.023) |
| β | 0.056***  (0.005) | -0.218***  (0.033) | 0.525***  (0.019) |
| γ | 0.007  (0.006) | 0.008  (0.036) | 0.045**  (0.021) |
| δ | -0.001  (0.006) | -0.039  (0.037) | -0.019  (0.021) |

Table S3.

Overview of how the distributions of types across treatments.

|  | Type 1  mildly altruistic/ selfish | Type 2 spiteful | Type 3  aheadness averse/ positively reciprocal |
| --- | --- | --- | --- |
| Overall | 28.03% | 31.15% | 40.82% |
| Treatments |  |  |  |
| Fellow Human | 16.11% | 10.74% | 73.15% |
| Programmer behind Machine | 27.33% | 20.67% | 52.00% |
| Token Player behind Machine | 26.67% | 15.33% | 58.00% |
| Machine Earns | 36.00% | 41.33% | 22.67% |
| Nobody Earns | 31.33% | 55.33% | 13.33% |
| No Information | 30.67% | 43.33% | 26.00% |

Table S4.

Distribution of age, gender, and stated opinion about algorithms across treatments.

|  | Age | % Female | Familiarity with new technology | Confidence  in new technology | Redistribution preferences |
| --- | --- | --- | --- | --- | --- |
| Treatments |  |  |  |  |  |
| Fellow Human | 35.48 | 53.02% | 2.30 | 2.54 | 3.02 |
| Programmer behind Machine | 37.07 | 54.00% | 2.31 | 2.61 | 2.87 |
| Token Player behind Machine | 34.86 | 52.67% | 2.20 | 2.53 | 3.09 |
| Machine Earns | 37.61 | 55.33% | 2.19 | 2.43 | 2.90 |
| Nobody Earns | 35.12 | 55.33% | 2.08 | 2.45 | 2.93 |
| No Info | 34.35 | 58.00% | 2.29 | 2.55 | 2.82 |

**Note**. *p*-values from pairwise Tukey’s range test for equality of means, all *p*s >0.10.

Table S5.

Distribution of employment status across treatments.

|  | Employment Status | | |
| --- | --- | --- | --- |
|  | Full-time | Part-time | Unemployed |
| Treatments |  |  |  |
| Fellow Human | 61.07% | 14.09% | 24.83% |
| Programmer behind Machine | 61.33% | 12.67% | 26.00% |
| Token Player behind Machine | 52.67% | 21.33% | 26.00% |
| Machine Earns | 57.33% | 16.67% | 26.00% |
| Nobody Earns | 56.67% | 18.67% | 24.67% |
| No Info | 51.33% | 19.33% | 29.33% |

**Note.** *p*-values from pairwise ꭕ^2^-test for equality of distributions all *p*s > 0.11.

**Table S6.**

Distribution of highest obtained degrees of education across treatments.

|  | **Highest obtained degree of education** | | | | |
| --- | --- | --- | --- | --- | --- |
|  | **No Degree** | **High School** | **Bachelor** | **Master** | **Ph.D.** |
| Treatments |  |  |  |  |  |
| Fellow Human | 1.34% | 35.57% | 43.62% | 16.78% | 2.68% |
| Programmer behind Machine | 0.67% | 34.00% | 46.67% | 18.00% | 0.67% |
| Token Player behind Machine | 1.33% | 40.00% | 44.67% | 13.33% | 0.67% |
| Machine Earns | 0.67% | 32.67% | 51.33% | 12.67% | 2.67% |
| Nobody Earns | 0.67% | 36.00% | 46.67% | 14.67% | 2.00% |
| No Info | 1.33% | 34.67% | 47.33% | 14.00% | 2.67% |

**Note**. *p*-values from pairwise ꭕ^2^-test for equality of distributions all *p*s > 0.41.

Table S7.

Regression table depicting the relationship between demographic information, stated opinion about algorithms, and preferences for redistribution on all four social preference parameters.

|  | **Social Preference Parameters** | | | |
| --- | --- | --- | --- | --- |
|  | **α** | **β** | **γ** | **δ** |
| Age | -0.0010  (0.0007) | 0.0008  (0.0008) | 0.0012  (0.0009) | 0.000  (0.0007) |
| Female | -0.0548***  (0.016) | 0.0052  (0.0185) | 0.0045  (0.019) | -0.0125  (0.0157) |
| Degree | 0.0102  (0.0106) | -0.0176 (0.0123) | -0.0181  (0.0128) | 0.0014  (0.0106) |
| Employment | -0.0043  (0.0097) | -0.0262**  (0.0112) | -0.0074  (0.0119) | 0.0016  (0.0098) |
| Familiarity | 0.0072  (0.0081) | -0.0011 (0.0094) | -0.0033  (0.0099) | 0.0022  (0.0082) |
| Confidence | 0.0118  (0.0089) | 0.0242**  (0.0103) | 0.0019  (0.0107) | 0.0139  (0.0089) |
| Redistribution | -0.0053  (0.0073) | 0.0202**  (0.0084) | -0.0030  (0.0092) | 0.0083  (0.0076) |
| Human behind Machine Treatments | 0.0214  (0.0230) | -0.1022*** (0.0267) | -0.0678**  (0.0273) | -0.0379*  (0.0226) |
| Machine Treatments | -0.0390*  (0.0220) | -0.3110***  (0.0255) | -0.1607***  (0.0262) | -0.1501***  (0.0216) |
| Constant | -0.0436  (0.0474) | 0.2593***  (0.055) | 0.1464**  (0.0575) | 0.0373  (0.0475) |
| N | 734 | 734 | 494 | 494 |
| R² | .0508316 | .2427405 | .0935331 | .1494835 |

**Note.** Regression of estimated parameters of social preferences on demographics and treatments. “Human behind Machine Treatments” comprises the Programmer behind Machine and the Token Player behind Machine treatments; “Machine Treatments” comprise the Machine Earns, Nobody Earns, and No Information treatments. * *p* < 0.10 ** *p* < 0.05 *** *p* < 0.01

Appendix for Social Preferences Towards Humans and Machines

Methods

***Systematic review of the instructions used in previous research.***

We asked a research assistant (RA3) to review all instructions of studies using strategic human–computer interactions identified in a recent large-scale review of over 160 studies ([March, 2021](#bib45)). Without providing a priori coding categories, we asked RA3 to develop an emerging coding scheme with the team of authors in an iterative feedback process. Together we identified six categories of instructions used in the existing research based on the implementations of the payoffs and the information provided, which served as the motivation for the experimental treatments (see Figure 2).

Dictator games

We used thirty versions of the dictator game. Figure S1 illustrates the payoff space for the choices participants faced. The games differed with regard to the absolute payoff for the dictator and the receiver. Note that option X is the dominant choice if a dictator solely cares about their own payoffs.

Reciprocity games

In a similar fashion to the dictator game, we used thirty versions of the reciprocity game. The first mover makes a binary decision between choosing an outside option Z (= unkind choice) and delegating the decision to the second mover (= kind choice). The second mover, in turn, decides whether to show positive (X) or negative reciprocity (Y). Using the strategy method, participants indicated what they would do for each game, knowing that the choice could be implemented. The resulting payoff space of the game is illustrated in Figure S2.

Machine play

The artificial human was generated using a normal distribution for age with mean and variance as observed in the Fellow Human subject population. For all other categorical variables, we used a simple discrete distribution with probability weights equal to the relative frequency among the subjects. As the classification algorithm, we used a random forest with 100 trees and an 80:20 train-test split. We achieve an accuracy of 81.83%, a precision of 81.02%, a recall of 80.11%, and an F_1_ score of 0.8026.

Coding procedure of open answers in the no information treatment

We asked a research assistant (RA1), who was naive to the purpose of the study, to read and code the open text field answers in the No Information treatment. Namely, we provided a document with the answers that participants provided to the question, “Where do you think the payoffs for the machine go?”. The research assistant was asked to code it according to the following scheme: Based on the answer given by the participant it appears that the participant believes that the money goes to (i) the programmer or company behind the algorithm; (ii) another uninvolved person; (iii) no one, the money is not paid out; (iv) earned by the machine (to use it further); (v) the experimenter; or (vi) another uninvolved third party.

Results of this coding are reported in Figure S3. In treatments Fellow Human, Programmer behind Machine, Token Player behind Machine, and Nobody Earns (Panels A, B, C, and E, respectively), the vast majority of participants report beliefs that are in line with the introduction shown at the beginning of the experiment. In the Machine Earns treatment, beliefs cluster around the two responses that no one, or the machine itself earns the payoffs. We analyze the participants’ answers in the No Information treatment in greater detail in the main text and Figure 7.

Results

Systematic coding of instructions

The descriptive statistics of the different payoff implementations and information provisions used in previous research are depicted in Figure S4. The figure shows that providing no information is the most frequently used implementation.

Efficiency concerns

When option X is—in addition to yielding a higher payoff for the dictator—also the efficient option, it is chosen in more than 90% of cases in all treatments. When both options have the same sum of payoffs, efficiency concerns can be neglected, and other motives, such as altruism, determine behavior. Here, when there is no human beneficiary, option X is still chosen in more than 90% of cases, while, when there is a human beneficiary, the share of option Y rises to about 20–25%. When option Y is the efficient option, even though option X maximizes the dictator’s payoffs, both concerns for altruism and efficiency favor choosing option Y. Option X is chosen in around 50% of all rounds in the Fellow Human treatment. It is selected significantly more often in the two machine treatments with a human beneficiary (around 60%, *p* < 0.01 in both cases) and even more often in machine treatments without a human beneficiary or without information than in those with a human beneficiary or in the Fellow Human treatment (between 78% and 86%, *p* < 0.01 in all cases).

Additional analysis on social preference parameters

The results add nuance to the aggregate results and confirm that cooperating with machines without a human beneficiary elicits lower social preferences than cooperation with humans and humans behind machines. Namely, participants show stronger behindness aversion when cooperating with machines, especially when no human behind the machine earns the payoffs (see the downward shift of yellow squares in Figure S5). Regarding reciprocity, we see a decrease in positive and negative reciprocity when participants cooperate with machines and no human earns the payoffs (see the leftward and downward shift of yellow squares in Figure S6).

Alternative specification of social preferences

As additional evidence on how the different treatments affect revealed social preferences, we measure parameters of another well-established model of distributional preferences by [Andreoni and Miller (2002).](#bib2) We run a structural estimation of the following CES utility (following [Fisman et al., 2015](#bib25)):

$$u_{1}=[(1-\varphi){\pi_{1}}^{\rho}+\varphi{\pi_{2}}^{\rho}]^{1/\rho}$$

The parameter 𝜑 is the weight put on the receiver’s payoffs and describes general fair-mindedness. If 𝜑=0, the individual is acting entirely selfish, and if 𝜑 = 0.5, s/he is fair-minded by weighing both players’ payoffs equally. The parameter 𝜌 describes the equality–efficiency trade-off. If 𝜌 > 0, the individual has distributional preferences towards maximizing efficiency, that is, the sum of both payoffs. If, however, 𝜌 < 0, s/he seeks to minimize inequity, that is, the difference between both payoffs.

The pattern for 𝜑 is similar to the one observed for parameters 𝛽, 𝛾, 𝛿 of the Charness-Rabin model (see Figure S7, Panel A). In the Fellow Human and the two treatments with a human beneficiary, we estimate a positive average 𝜑, that is, a positive weight on the receiver’s payoffs. Almost all comparisons of the human treatments with the machine treatments, without a human beneficiary or with no information, where the average 𝜑 is negative, are significant (except for Programmer vs. Machine Earns and Programmer vs. No Info).

Regarding the equality–efficiency trade-off, the average estimated parameter 𝜌 is negative for all treatments (see Figure S7, Panel B). This speaks in favor of inequity-minimizing rather than efficiency-maximizing choices. Further, there are no significant differences in 𝜌 across all treatments except for the Fellow Human treatment, where the average subject exhibits stronger fairness concerns, and 𝜌 is more negative compared to the value in the Nobody Earns treatment (*p* = 0.03).

How individual factors shape social preferences

To test whether the random allocation to the different treatments succeeded, we examine the distribution of the assessed demographic information of age and gender, as well as the stated familiarity with and confidence in new technology, and the redistribution preferences across all treatments. Confirming successful randomization, the analyses reveal no significant differences across treatments (see Table S4). Nor does the employment status (see Table S5) or the highest obtained education degree of participants (Table S6) differ across treatments.
